# Direct oral anticoagulants versus vitamin K antagonists in patients with atrial fibrillation and cancer a meta-analysis

**DOI:** 10.1007/s11239-020-02304-3

**Published:** 2020-10-12

**Authors:** Marco Valerio Mariani, Michele Magnocavallo, Martina Straito, Agostino Piro, Paolo Severino, Gino Iannucci, Cristina Chimenti, Massimo Mancone, Domenico Giovanni Della Rocca, Giovanni Battista Forleo, Francesco Fedele, Carlo Lavalle

**Affiliations:** 1 Department of Cardiovascular, Respiratory, Nephrology, Anesthesiology and Geriatric Sciences, Sapienza University of Rome, Viale del Policlinico 155, 00161 Rome, Italy; 2 Department of Translational and Precision Medicine, Sapienza University of Rome, Rome, Italy; 3 Texas Cardiac Arrhythmia Institute, St. David’s Medical Center, Austin, TX USA; 4 Department of Cardiology, Luigi Sacco Hospital, Milan, Italy

**Keywords:** Atrial fibrillation, Cancer, Direct oral anticoagulants, Vitamin K antagonists

## Abstract

**Background:**

Direct oral anticoagulants (DOACs) are recommended as first-line anticoagulants in patients with atrial fibrillation (AF). However, in patients with cancer and AF the efficacy and safety of DOACs are not well established.

**Objective:**

We performed a meta-analysis comparing available data regarding the efficacy and safety of DOACs vs vitamin K antagonists (VKAs) in cancer patients with non-valvular AF.

**Methods:**

An online search of Pubmed and EMBASE libraries (from inception to May, 1 2020) was performed, in addition to manual screening. Nine studies were considered eligible for the meta-analysis involving 46,424 DOACs users and 182,797 VKA users.

**Results:**

The use of DOACs was associated with reduced risks of systemic embolism or any stroke (RR 0.65; 95% CI 0.52–0.81; p 0.001), ischemic stroke (RR 0.84; 95% CI 0.74–0.95; p 0.007) and hemorrhagic stroke (RR 0.61; 95% CI 0.52–0.71; p 0.00001) as compared to VKA group. DOAC use was associated with significantly reduced risks of major bleeding (RR 0.68; 95% CI 0.50–0.92; p 0.01) and intracranial or gastrointestinal bleeding (RR 0.64; 95% CI 0.47–0.88; p 0.006). Compared to VKA, DOACs provided a non-statistically significant risk reduction of the outcomes major bleeding or non-major clinically relevant bleeding (RR 0.94; 95% CI 0.78–1.13; p 0.50) and any bleeding (RR 0.91; 95% CI 0.78–1.06; p 0.24).

**Conclusions:**

In comparison to VKA, DOACs were associated with a significant reduction of the rates of thromboembolic events and major bleeding complications in patients with AF and cancer. Further studies are needed to confirm our results.

**Electronic supplementary material:**

The online version of this article (10.1007/s11239-020-02304-3) contains supplementary material, which is available to authorized users.

## Highlights

- Anticoagulation in patients with atrial fibrillation and malignances is challenging due to cancer-related factors.
- The efficacy and safety of direct oral anticoagulants in cancer patients is not well established. In our meta-analysis the use of direct oral anticoagulants was associated with reduced risk of ischemic and hemorragic stroke, major bleedings and intracranial and gastrointestinal bleedings in comparison to vitamin K antagonists.
- Direct oral anticoagulants use was related to more effective and safer profile as compared to vitamin K antagonists and may represent a suitable anticoagulant strategy in cancer patients with atrial fibrillation.

## Introduction

Atrial fibrillation (AF) is the most commonly diagnosed clinical arrhythmia and its prevalence increases with age, up to 18% at 85 years of age [1]. AF confers an increased risk of cardiovascular complications, including a fivefold risk of thromboembolic events, as such stroke and transient ischemic attack (TIA) [2], therefore anticoagulant therapy is recommended on the basis of individual thrombotic risk determined by CHA2DS2VASc risk score [3]. Given the high prevalence of malignances and AF in the elderly, the progressive aging of population will probably lead to an increased prevalence of cancer in AF patients. Currently, up to 25% of AF population has comorbid cancer [4]. On the other hand, AF is commonly diagnosed in cancer patients and may be related to shared comorbid states, inflammation, direct tumor effect, complications of cancer surgery or anti-cancer therapy [5–8]. Anticoagulant management of AF population with cancer is challenging because of an the increased propensity for both thrombosis and bleeding of this population [7]. As a result, the search for an acceptable anticoagulation treatment is a major clinical issue, currently unsolved. Direct oral anticoagulants (DOACs) have been demonstrated non-inferior to vitamin K antagonists (VKAs) for stroke prevention in non-valvular AF patients [9–12] with even better safety profile. Therefore, current European Heart Rhythm Association guidelines [13] recommend DOACs over VKAs as preferred anticoagulation strategy in patients with AF who are eligible for DOAC therapy. However, these recommendations cannot be extended to AF patients with malignances because in randomized clinical trials (RCTs) of DOACs for stroke and systemic embolism prevention in AF, cancer patients were underrepresented. Nevertheless, post-hoc analyses of RCTs of DOACs [14–17] and retrospective population or cohort studies [18–23] have shown promising results of DOACs compared to VKAs in non-valvular AF patients with cancer. Therefore, we aimed to systematically assess the available evidences in the literature regarding the safety and efficacy of DOACs in comparison to VKAs in patients affected by non-valvular AF and cancer.

## Methods

### Search strategy, selection criteria and outcomes

The present meta-analysis was performed according to Cochrane Collaboration and Preferred Reporting Items for Systematic Reviews and Meta-Analyses (PRISMA) statements [24].

An online search of Pubmed, Cochrane Registry, Web of Science, Scopus and EMBASE libraries (from inception to May, 1 2020) was performed, in addition to manual screening. We used the following keywords: [(atrial fibrillation) OR (non-valvular atrial fibrillation)]; [(neoplasia) OR (neoplasm) OR (cancer) OR (malignancy) OR (tumor) OR (leukemia) OR (lymphoma)]; [(non-vitamin K antagonists) OR (new oral anticoagulants) OR (novel oral anticoagulants) OR (direct oral anticoagulants) OR (direct thrombin inhibitors) OR (oral thrombin inhibitors) OR (factor Xa inhibitors) OR (NOACs) OR (DOACs) OR (dabigatran) OR (rivaroxaban) OR (apixaban) OR (edoxaban)]; [(vitamin K antagonists) OR (warfarin) OR (VKAs)]. No language restriction was applied.

Studies on patients with non-valvular AF and cancer with the following characteristics were considered eligible for the meta-analysis: (1) RCTs and post-hoc analysis of RCTs, (2) non-randomized prospective or retrospective cohort studies comparing any DOACs (apixaban, rivaroxaban, edoxaban and dabigatran) at any dose vs VKAs for stroke prevention, (3) if results on efficacy and/or safety of DOACs vs VKAs in non-valvular AF patients with malignances were clearly reported. Reviews, editorials, letters, meta-analysis, case reports and abstracts were excluded.

We evaluated the following efficacy outcomes: thromboembolic events, including any type of stroke or systemic embolism (SSE), ischemic stroke, hemorrhagic stroke, myocardial infarction (MI), all-cause mortality and cardiovascular mortality. Among safety outcomes we included: major bleeding, non-major clinically relevant bleeding (NMCRB), any intracranial or gastrointestinal bleeding and any bleeding (including major bleeding, non-major clinically relevant bleeding and minor bleeding). Major bleeding events were defined in accordance with the criteria of the International Society of Thrombosis and Haemostasis (ISTH) [25], whereas non-major clinically relevant major bleedings were defined as any bleeding that does not fit the criteria for the ISTH definition of major bleeding but does meet at least one of the following criteria: (1) requiring medical intervention by a healthcare professional; (2) leading to hospitalization or increased level of care; (3) prompting a face to face evaluation.

Two independent reviewers (MVM and AP) screened all abstracts and titles to identify potentially eligible studies, of which full text was subsequently interrogated. Agreement of the two reviewers was required for eligibility of studies for analysis. Disagreements regarding the inclusion or the classification of a study were solved by a third reviewer (CL).

### Data extraction and quality assessment

Data extraction was performed by two reviewers (MVM and AP). For each study the following data were collected: first author and year of publication, study design, type of DOAC, population size, number of DOAC/VKA users, efficacy outcomes, safety outcomes, cancer types, propensity-score-matched risk ratios (RRs) or adjusted RRs, or unadjusted RRs and corresponding 95% confidence intervals (CIs). In studies reporting outcomes of different dosages of the same DOAC, we pooled data to calculate the combined RRs.

Study quality was formally evaluated by two reviewers (MVM and AP) using the Newcastle–Ottawa scale for post-hoc analysis of RCTs and cohort studies [26]. Three categories were included in the analysis, with some of them having subcategories for assessment. Studies were subsequently classified into one of three categories: (i) high quality: 6–9 points; (ii) satisfactory quality: 3–5 points; and (iii) unsatisfactory quality: 0–2 points [26].

### Statistical analysis

Descriptive statistics is presented as means and standard deviations (SD) for continuous variables or number of cases (n) and as percentages (%) for dichotomous and categorical variables. Statistical analysis was performed using Review Manager (RevMan version 5.3, the Cochrane Collaboration, 2014; Oxford, United Kingdom). Statistical heterogeneity on each outcome of interest was quantified using I^2^ statistic and the Cochrane Q test. Values of I^2^ statistic, ≤ 25%, 50%, and ≥ 75% indicated low, moderate, and high heterogeneity, respectively, whereas for Q statistic, substantial heterogeneity was defined as a p < 0.1. Data were pooled using a random effect model in consideration of the expected heterogeneity among studies. For each study, the effect estimates chosen were the RRs and their corresponding 95% CIs, which were converted to their corresponding natural logarithms and standard errors. Sensitivity analysis was performed evaluating the effect of single study withdrawal on the pooled RR for each outcome. In addition, we also performed subgroup analyses based on the design of the study, the presence of propensity-score matched or adjusted RRs and we separately analyzed patients with active cancer to investigate the efficacy and safety of DOACs in comparison to VKAs in this setting. A P value ≤ 0.05 was considered statistically significant.

## Results

### Study selection, quality of evidence and patients characteristics

**Fig. 1:**
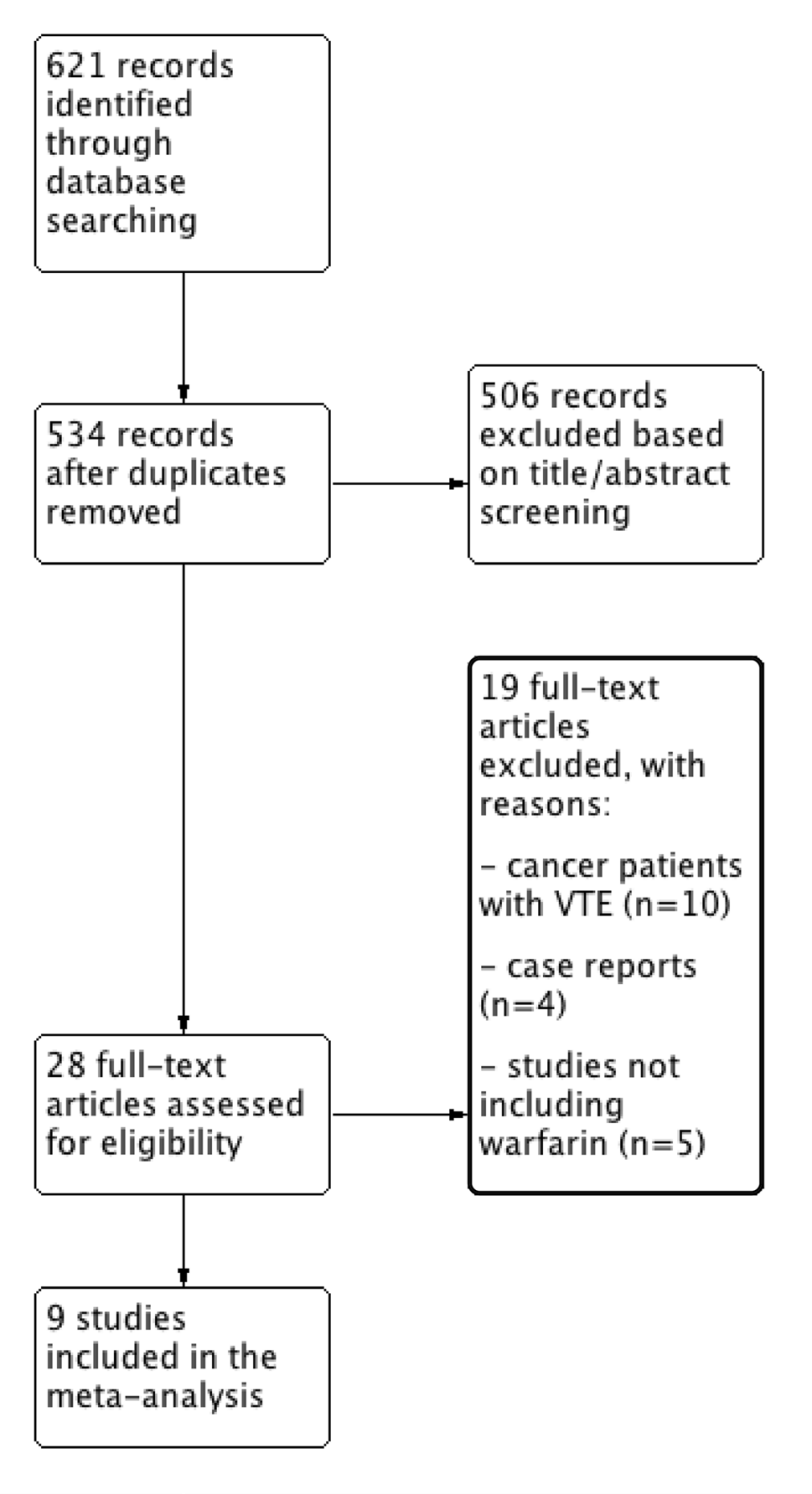
Study flow diagram. *VTE* venous thromboembolism

The literature search process identified 621 studies (Fig. 1). After excluding duplicate publications, reviews, editorials, letters, meta-analysis, case reports and abstracts, 28 studies were fully reviewed and 9 studies were considered eligible for the meta-analysis. In particular, the studies used for the analysis included 3 post-hoc analysis from RCTs [14–16] and 6 retrospective population-based cohort studies [18–23] involving 46,424 DOACs users and 182,797 VKA users (Table 1).

**Table 1:** Baseline characteristics of the studies

Author	Study type	DOACs tested	DOAC/Warfarin users (n)	Patients (n)	Efficacy outcomes	Safety outcomes	Patients with active cancer (n)	Mean follow-up (y)	NOS score
Melloni 2017 [16]	Post hoc analysis from ARISTOTELE trial	Apixaban	615/621	1236	SSE, MI, all-cause death	Major bleeding (ISTH criteria),NMCR bleeding, any bleeding^a^	157	1.8	9
Ording 2017 [20]	Retrospective population-based cohort study	Dabigatran, apixaban, rivaroxaban	1809/10046	11855	SE, ischemic stroke, hemorragic stroke, MI, VTE	Gastrointestinal bleeding, lung and urinary bleeding	N.A	1	7
Kim 2018 [19]	Retrospective population-based cohort study	Dabigatran, apixaban, rivaroxaban	388/388	776	SSE, ischemic stroke, all-cause death	Major bleeding (ISTH criteria), gastrointestinal bleeding, intracranial bleeding, other bleeding	776	1.8	8
Fanola 2018 [15]	Post hoc analysis from ENGAGE AF-TIMI 48 trial	Edoxaban	395/758	1153	SSE, ischemic stroke, MI, all- cause death, cardiovascular death	Major bleeding (ISTH criteria), gastrointestinal bleeding, NMCR bleeding, any bleeding^a^	1153	2.8	9
Shah 2018 [18]	Retrospective population-based cohort study	Dabigatran, apixaban, rivaroxaban	6075/10 021	16096	Ischemic stroke, VTE	Severe bleeding (intracranial or gastrointestinal), other bleeding	16096	1	8
Chen 2019 [14]	Post hoc analysis from ROCKET AF trial	Rivaroxaban	Efficacy: 307/329 Safety: 309/331	640	SSE, ischemic stroke, hemorrhagic stroke, MI, VTE, all-cause death, cardiovascular death	Major bleeding (ISTH criteria), intracranial bleeding, NMCR bleeding, any bleeding^a^	50	1.9	8
Sawant 2019 [21]	Retrospective population-based cohort study	Dabigatran, apixaban, rivaroxaban	36340/160177	196517	Ischemic stroke and hemorragic stroke	None	N.A	1	6
Yasui 2019 [23]	Retrospective single-center cohort study	Dabigatran, apixaban, rivaroxaban, edoxaban	127/97	224	SSE, ischemic stroke	Major bleeding (ISTH), and gastrointestinal and Intracranial major bleeding	224	1	7
Wu 2020 [22]	Retrospective population-based cohort study	Dabigatran, apixaban, rivaroxaban, edoxaban	366/366	732	SSE, MI, all-cause death	Major bleeding (ISTH) and gastrointestinal major bleeding	732	1	8

*DOACs* direct oral anticoagulants, *ISTH* International Society of Hemostasis and Thrombosis, *MI* myocardial infarction, *NMCR* non-major clinically relevant, *NOS* Newcastle–Ottawa Scale, *SE* systemic embolism, *SSE* stroke or systemic embolism, *VTE* venous thromboembolism

^a^Any bleedings includes major, NMCR and minor bleedings

The post-hoc analysis from ROCKET AF [14], ARISTOTLE [16] and ENGAGE AF-TIMI 48 [15] trials reported the efficacy and safety of rivaroxaban, apixaban and edoxaban, respectively, versus VKA in patients with a history of cancer. Among the retrospective population-based cohort studies, 4 out 6 reported the management of non-valvular AF in cancer patients taking apixaban, rivaroxaban and dabigatran [18–21]. The remaining retrospective studies Wu et al. [22] and Yasui [23] included apixaban, rivaroxaban, dabigatran and edoxaban in the analysis.

The more prevalent malignances across the studies were gastrointestinal, breast and prostatic cancers (Supplemental Material, Table S1). All the studies but three reported the propensity-score-matched or adjusted RRs [20, 21, 23] for pre-specified outcomes. In 7 out 9 studies, major bleedings were considered according to the ISTH criteria [24], whereas one study [20] reported any diagnosis of gastrointestinal, lung and urinary bleedings as safety outcomes. Three out 9 studies reported data on time-in-therapeutic range (TTR) [14, 15, 19]. Mean follow-up was at least 1 year for all included studies. Seven out 9 studies reported data about cancer status, while Ording et al. [20] and Sawant et al. [21] did not distinguish active cancer form remote cancer history. In particular, active cancer was defined differently across studies as newly diagnosed cancer during study period or diagnosed within last 6 months, and/or as actively treated cancer with ongoing cancer therapy (chemotherapy, radiotherapy or surgery) or treated during the previous 6 months or year, and/or newly diagnosed neoplasm at imaging (Supplemental material, Table S2).

All the studies included in the analysis had a moderate-to-high quality as indicated by a Newcastle–Ottawa Scale score > 6 (Table 1).

### Efficacy and safety of DOACs vs VKAs in cancer patients with AF

Table2 summarizes the RRs and 95% CI for study outcomes. The use of DOACs was associated with reduced risks of SSE (RR 0.65; 95% CI 0.52–0.81; p 0.001), ischemic stroke (RR 0.84; 95% CI 0.74–0.95; p 0.007) and hemorrhagic stroke (RR 0.61; 95% CI 0.52–0.71; p 0.00001) as compared to VKA group. No statistically significant differences were found among DOACs and VKAs regarding the risks of cardiovascular mortality (RR 0.76; 95% CI 0.53–1.09; p 0.14), all-cause mortality (RR 0.84; 95% CI 0.59–1.20; p 0.34) and myocardial infarction (RR 0.71; 95% CI 0.48–1.04; p 0.08).

**Table 2:** Efficacy and Safety of DOACs vs VKAs in patients with cancer and atrial fibrillation

Outcome	Random-effects model		Fixed-effects model		Retrospective cohorts		Post-hoc analyses		Propensity-score matched cohorts		Active cancer cohorts	
	RR and 95% CI	p-value	RR and 95% CI	p-value	RR and 95% CI	p-value	RR and 95% CI	p-value	RR and 95% CI	p-value	RR and 95% CI	p-value
SSE	0.65 (0.52–0.81)	0.0001	0.68 (0.59–0.79)	< 0.00001	0.61 (0.49–0.80)	0.0004	0.76 (0.54–1.07)	0.11	0.53 (0.32–0.86)	0.01	0.46 (0.27–0.78)	0.004
Ischemic Stroke	0.84 (0.74–0.95)	0.007	0.90 (0.88–0.93)	< 0.00001	0.85 (0.75–0.97)	0.01	0.78 (0.38–1.58)	0.49	0.65 (0.42–1.01)	0.06	0.63 (0.40–0.99)	0.05
Hemorragic Stroke	0.61 (0.52–0.71)	0.00001	0.61 (0.54–0.69)	0.00001	0.61 (0.51–0.73)	0.00001	0.36 (0.04–3.24)	0.36	0.36 (0.04–3.24)	0.36	N.A	N.A
MI	0.71 (0.48–1.04)	0.08	0.69 (0.49–0.96)	0.03	0.81 (0.21–3.11)	0.76	0.78 (0.51–1.19)	0.25	0.84 (0.56–1.26)	0.40	0.91 (0.34–2.43)	0.85
CV Death	0.76 (0.53–1.09)	0.14	0.76 (0.53–1.09)	0.14	N.A	N.A	0.76 (0.53–1.09)	0.14	0.76 (0.53–1.09)	0.14	0.82 (0.53–1.27)	0.37
All-cause Death	0.84 (0.59–1.20)	0.34	0.92 (0.81–1.04)	0.18	0.66 (0.30–1.43)	0.29	1.01 (0.73–1.09)	0.96	0.84 (0.59–1.20)	0.34	0.74 (0.48–1.14)	0.17
MB	0.68 (0.50–0.92)	0.01	0.82 (0.71–0.96)	0.01	0.53 (0.33–0.94)	0.03	0.83 (0.65–1.06)	0.13	0.67 (0.49–0.92)	0.01	0.62 (0.42–0.90)	0.01
MB or CRNMB	0.94 (0.78–1.13)	0.50	0.94 (0.78–1.13)	0.50	N.A	N.A	0.94 (0.78–1.13)	0.50	0.94 (0.78–1.13)	0.50	0.75 (0.52–1.07)	0.11
IC or GI Bleeding	0.64 (0.47–0.88)	0.006	0.83 (0.71–0.97)	0.02	0.64 (0.44–0.92)	0.02	0.30 (0.05–1.68)	0.17	0.58 (0.39–0.87)	0.008	0.65 (0.46–0.94)	0.02
Any Bleeding	0.91 (0.78–1.06)	0.24	0.92 (0.83–1.02)	0.10	0.87 (0.64–1.18)	0.37	0.93 (0.74–1.18)	0.56	0.95 (0.81–1.12)	0.55	0.95 (0.81–1.12)	0.53

The RRs for each outcome obtained by pooling data using a random-effects model and different sensitivity analyses are shown

*CI* confidence interval, CV cardiovascular, *IC* intracranial, *GI* gastrointestinal, *MI* myocardial infarction, *MB* major bleeding, *NMCRB* non-major clinically relevant bleeding, *RR* relative risk, *SSE* stroke or systemic embolism

**Fig. 2:**
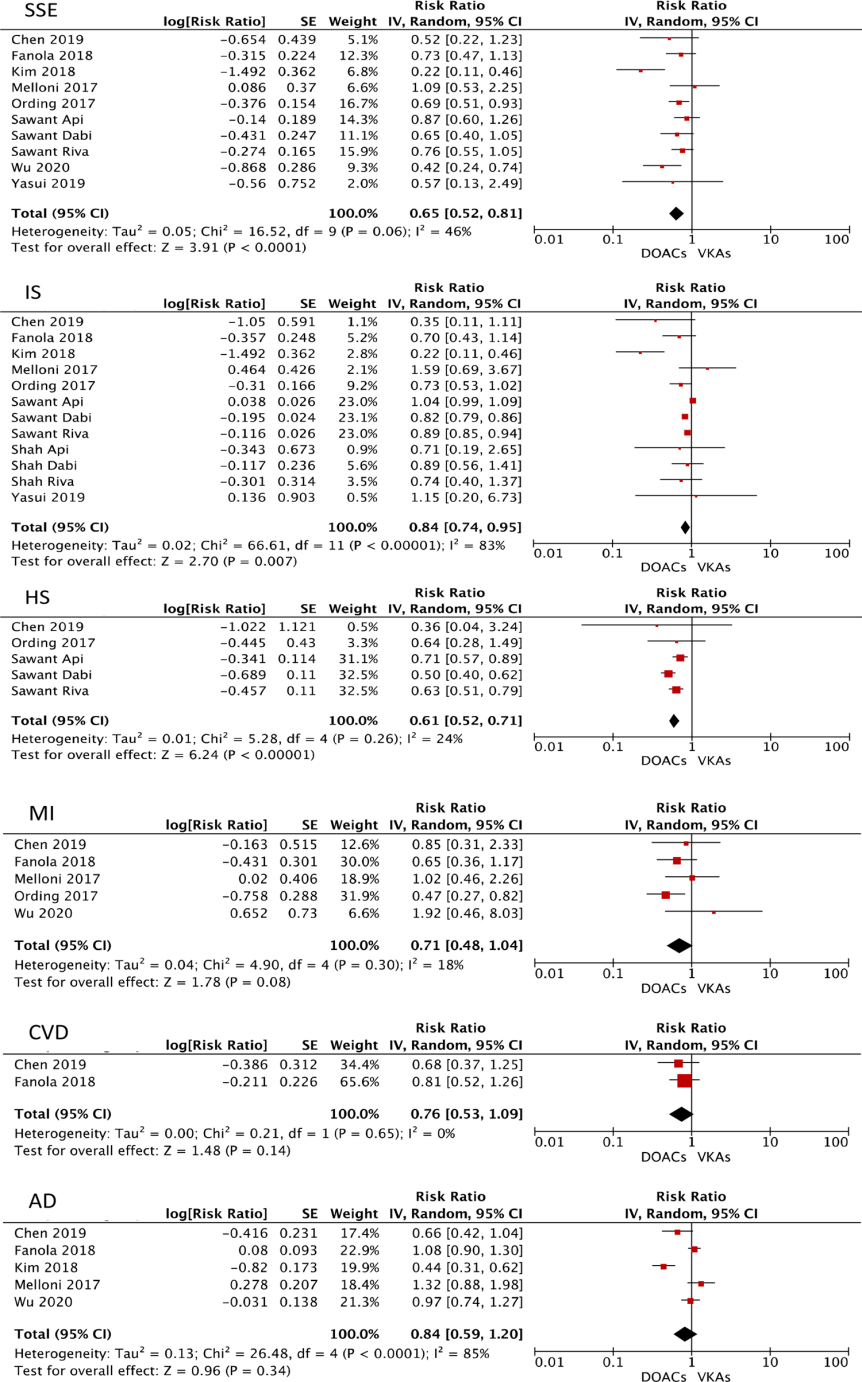
Forest plots showing the comparison between DOACs vs VKAs in patients with cancer and AF. The RRs for efficacy outcomes are shown *AD* all-cause death, *CVD* cardiovascular death, *DOAC* direct oral anticoagulant, *HS* hemorrhagic stroke, *IS* ischemic stroke, *MI* myocardial infarction, *SSE* stroke or systemic embolism

**Fig. 3:**
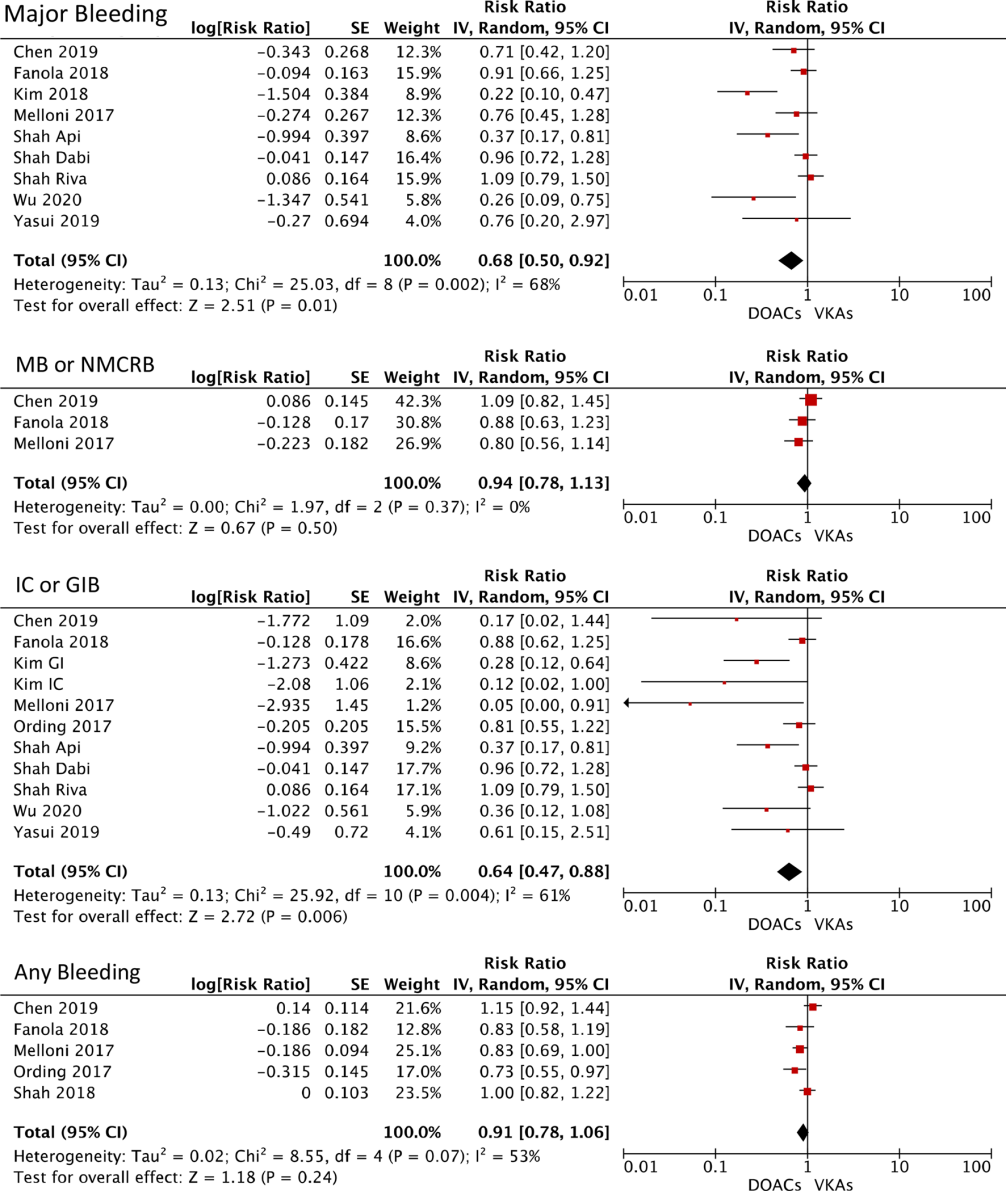
Forest plots showing the comparison between DOACs vs VKAs in patients with cancer and AF. The RRs for safety outcomes are shown *GIB* gastrointestinal bleeding, IC intracranial; MB: major bleeding, *NMCRB* non-major clinically relevant bleeding

In comparison to VKAs, DOACs was related to a statistically significant reduction of the risks of major bleeding (RR 0.68; 95% CI 0.50–0.92; p 0.01) and intracranial or gastrointestinal bleeding (RR 0.64; 95% CI 0.47–0.88; p 0.006). However, DOACs provided a non-statistically significant risk reduction of the outcomes major bleeding or NMCRB (RR 0.94; 95% CI 0.78–1.13; p 0.50) and any bleeding (RR 0.91; 95% CI 0.78–1.06; p 0.24) compared to VKAs. Forest plots regarding efficacy and safety outcomes are shown in Fig. 2 and Fig. 3, respectively.

### Sensitivity analysis

Sensitivity analysis was performed evaluating the effect of single study withdrawal on the pooled RR for each outcome. The use of DOACs was consistently associated with decreased risk of SSE, ischemic stroke and hemorrhagic stroke also after excluding each study in turn. The RRs for each outcome did not change when calculated with a fixed-effect model-based analysis (Table 2). Similar rates of efficacy and safety outcomes were obtained when pooling the data derived from the 6 retrospective population-based cohort studies, whereas DOACs and VKAs yielded the same efficacy and safety in cancer patients with AF when pooling data from the 3 post-hoc analysis (Table 2). Noteworthy, the analysis including the 6 out 9 studies reporting propensity-score matched or adjusted RRs consistently showed that DOAC use was related to a significant reduction in the risk of SSE (RR 0.53; 95% CI 0.32–0.86; p 0.01), major bleeding (RR 0.67; 95% CI 0.49–0.92; p 0.01) and intracranial or gastrointestinal bleeding (RR 0.58; 95% CI 0.39–0.87; p 0.008), whereas a strong tendency towards risk reduction of ischemic stroke (RR 0.64; 95% CI 0.42–1.01; p 0.06) was found across DOAC group as compared to VKA group (Table 2). When considering patients with active cancer, DOACs were still associated with reduction of the rates of SSE (RR 0.46; 95% CI 0.27–0.78; p 0.004), ischemic stroke (RR 0.63; 95% CI 0.40–0.99; p 0.05), major bleeding (RR 0.62; 95% CI 0.42–0.90; p 0.01) and intracranial or gastrointestinal bleeding (RR 0.65; 95% CI 0.46–0.94; p 0.02) as compared to VKAs. The forest plots of sensitivity analyses are shown in Figure S1-S10 (Supplemental Material).

## Discussion

To our knowledge, this is the largest meta-analysis comparing efficacy and safety of DOACs versus VKAs in patients with malignancies. The main findings of our study are as follows: (1) DOACs use resulted in lower rates of any stroke or systemic embolism, as compared to VKAs use; (2) DOACs were associated with safer profile risk than VKAs, as the use of DOACs resulted in a statistically significant reduction of major bleedings and intracranial or gastrointestinal bleedings; (3) in comparison to VKAs, DOACs were found to be non-inferior for the outcomes MI, cardiovascular death, all-cause death, major bleeding or non-major clinically relevant bleeding and any bleeding.

The best anticoagulation management in cancer patients is still debated in consideration of the unique clinical risk profile carried by malignancies. Indeed, cancer patients have higher rates of venous thromboembolism (VTE) and arterial thrombosis for inflammatory cytokines, tumor vascular invasion and vasculotoxic cancer therapies, whereas cancer-related thrombocytopenia and chemotherapy-related bone marrow suppression increase bleeding complications [6–8]. As a result, in the past years concerns about bleeding complications and paucity of data have led to an underuse of DOACs in cancer patients with non-valvular AF. As reported by Ording et al. [20] from Danish population-based medical databases, only 15% of patients with cancer and AF are currently prescribed DOACs (vs. VKAs) in clinical daily practice. However, mounting evidences are demonstrating that DOACs could represent a valid choice in patients with cancer. Actually, Select-D Trial and Hokusai VTE Cancer trial respectively demonstrated that rivaroxaban and edoxaban were non-inferior to low-molecular-weight heparin (LMWH) in treatment of cancer-related VTE [28, 29], although at cost of increased bleeding complications. As a result, rivaroxaban and edoxaban are currently recommended for VTE treatment as alternative to LMWH in cancer patients with low gastrointestinal and genitourinary bleeding risk, low drug-drug interactions with DOACs and on the basis of patients’ preferences [30, 31]. The recently published ADAM VTE trial and Caravaggio Trial have shown the efficacy of apixaban as compared to dalteparin in the prevention of recurrence of cancer-related VTE, with similar bleeding rates among the study arms [32, 33]. Our meta-analysis is consistent with the effectiveness of DOACs in the management of cancer-related thrombosis. A previous meta-analysis by Deng et al. [34] found that DOACs were associated to statistically significant reduced rates of the composite outcome SSE, but no differences were found for the outcomes ischemic stroke. In line with previous report, in our analysis DOACs were related with lower rates of SEE, but in addition we found a significantly reduction of the outcomes ischemic stroke and hemorrhagic stroke in comparison to VKAs. Interestingly, better efficacy in preventing thromboembolic events was associated with a reduced risk of major bleeding or gastrointestinal and intracranial bleedings as compared to VKAs, while no differences where found for the outcomes major bleeding or clinically relevant non-major bleeding and any bleeding. An apparently better safety profile of DOACs in comparison to VKAs in cancer patients has never been clearly demonstrated so far, as in the meta-analysis by Deng et al. [34] DOACs showed borderline significant reduction of major bleeding and the reduction of gastrointestinal or intracranial bleeding was not consistent among different sensitivity analyses. Conversely, we found a statistically significant lower rates of major bleeding and gastrointestinal and intracranial bleeding that was consistent in all sensitivity analysis but one including only post-hoc analyses of RCTs. The better results obtained with DOACs vs VKAs on thromboembolic and bleeding events may be driven by different factors, as the pharmacodynamic and pharmacokinetic features of VKAs, whose anticoagulation activity relies on TTR. As previously reported by Kim et al. [35], the obtaining of an optimal range of international normalized ratio (INR) is difficult in patients with malignances receiving cancer therapy, so that the prevalence of patients with active cancer reaching a TTR > 60% during follow-up is as high as 10%. Although a suboptimal TTR during VKA therapy reduces anticoagulant activity and accounts for lower VKA efficacy, Kim et al. [19] showed that also in patients with optimal TTR DOACs still were more effective and safer than VKAs. Moreover, the benefits of DOACs over VKAs could be enhanced in patients with active cancer status, that has been defined in different ways across studies as a newly diagnosed cancer or a cancer receiving therapy during the study period or a cancer that received specific therapy within 6 months/1 year before starting anticoagulation (Supplemental material, Table S2). Patients with active cancer are more likely to undergo invasive anticancer treatment, such as surgery or biopsy, or pharmacologic anticancer therapy that may interact with anticoagulant drugs. Hence, active cancer is more likely associated to anticoagulant therapy interruption, reported as high as 69.2% by Fanola et al. [15] and 29% by Melloni et al. [16], for safety concerns about surgery or drug-drug interactions. In this setting, DOACs may offer advantages over VKAs in terms of both efficacy and safety outcomes, because of their short onset time, short half-life, low inter- and intra-individual variability and drug-drug interactions. Moreover, VKA interruption requires heparin bridging with increased risk of bleedings. The outcomes of DOACs in patients with active cancer have never been largely described so far, but as noted above, this is a high-risk population in which imbalance of thrombotic and bleeding risks may lead to serious outcomes. Hence, we performed a sensitivity analysis pooling data regarding efficacy and safety of DOACs vs VKAs only in patients with AF and active cancer and we showed for the first time in this peculiar population that DOACs use is related to stronger thromboembolic risk reduction and more favorable risk profile than VKAs, lowering the rates of the outcomes SSE, ischemic stroke, major bleeding and gastrointestinal or intracranial bleeding.

This meta-analysis is the largest comparing efficacy and safety of DOACs vs VKAs in patients with non-valvular AF and cancer so far, and clearly shows that DOACs may be considered a suitable anticoagulant agent in this challenging subgroup of patients. DOACs use was related to a more effective and safer profile as compared to VKAs and significantly lowered the rates of SSE, ischemic and hemorrhagic stroke, VTE, major bleeding and gastrointestinal and intracranial bleeding. In addition, DOACs represent a handy therapeutic strategy, not requiring frequent monitoring of INR and with less expected drug-drug interactions, providing a less burdensome alternative to a highly frail population. Moreover, our analysis confirms the favorable efficacy and safety profile of DOACs in cancer patients recently outlined by Giustozzi et al. in a meta-analysis of RCTs comparing safety and efficacy of DOACs vs LMWH in the treatment of cancer-related VTE [36, 37]. Although the choice of anticoagulant therapy should be tailored on patients’ preferences and bleeding risk profile, taken together these evidences are hypothesis-generating, suggesting that the use of DOACs may represent a reasonable choice in cancer patients with AF. Prospective randomized trials evaluating the efficacy and safety profiles of DOACs vs VKAs in cancer patients with AF are eagerly awaited, in order to give more confidence to physicians that are involved in clinical daily management of this troublesome population.

## Limitations

Our study presents some limitations. First, the meta-analysis only includes post-hoc analysis of RCT or retrospective population-based cohorts. The observational nature of reported data may affect the generalizability of our findings that should be considered as exploratory. However, prospective RCTs on this field are currently missing and we included in the analysis the best evidence produced so far. Second, all but three studies reported the adjusted RRs or propensity-score matched RRs. Including in the analysis unadjusted data for confounders may reduce the validity of the study. However, we performed a sensitivity analysis excluding the studies reporting only unadjusted RRs that confirmed the main findings of our study. Third, active cancer has been associated with worse outcomes [16, 27]. We performed a subgroup analysis on safety and efficacy of different anticoagulation strategies in patients with active cancer but the lack of information about type of cancer treatment and the heterogeneity among the studies regarding the definition of active cancer may have affected our results that need to be confirmed. Fourth, we did not perform an analysis on the basis of different cancer stages due to the lack of this information in the majority of the included studies. Fifth, TTR for VKA was reported only in few studies. The lack of TTR did not allow concluding that DOACs are superior to VKAs because a suboptimal TTR may have affected VKA safety and efficacy as shown by Kim et al. [19]. Finally, cancer population included in this meta-analysis is heterogeneous and some studies, as such the post-hoc analysis of ROCKET-AF trial [14] and the study by Sawant et al. [21], excluded patients with life-expectancy < 2 years and < 1 year, respectively, possibly with advanced cancer. However, the inclusion of large population-based, real-life studies in the analysis might have possibly overcome this pitfall.

## Conclusions

In patients with cancer and non-valvular AF, the use of DOACs is associated with a significant reduction of thromboembolic and bleeding events and this result is consistent among patients with active cancer. Prospective randomized studies are needed to confirm our findings and address gaps in evidence.

## Electronic supplementary material

Below is the link to the electronic supplementary material.

Supplementary file1 (docx 7980 kb)
