# Supplementary material for: Direct oral anticoagulants versus vitamin K antagonists in patients with atrial fibrillation and cancer a meta-analysis
Source: J Thromb Thrombolysis. 2020 Oct 12;51(2):419–29. doi: 10.1007/s11239-020-02304-3 (PMC7886836; doi:10.1007/s11239-020-02304-3)
Supplement: Supplementary file 1 — Supplementary file1 (docx 7980 kb) [file 11239_2020_2304_MOESM1_ESM.docx]

**Supplemental Material**

| **Author** | **Cancer Type** |
| --- | --- |
| **Melloni 2017 [16]** | Bladder (7%), breast (16%), colon (11%), gastric (2%), lung (3%), melanoma (6%), others (10%), ovarian/uterus (6%), prostate (29%), rectal (3%), renal cell carcinoma (4%), Hodgkin’s lymphoma (1%), leukemia (<1%), lymphoma (1%), Non- Hodgkin’s lymphoma (1%) |
| **Ording 2017 [20]** | Urological cancer was diagnosed in 15% of cancer patients, followed by breast cancer (12%), gastrointestinal cancer (12%), lung cancer (4%), hematological cancer (3%), and intracranial cancer (0.1%), other sites (54%) |
| **Kim 2018 [19]** | Stomach (20.6%), colorectal (14.9%), thyroid (10.8%), prostate (9.3%), lung (12.2%), melanoma (5.9%), biliary tract (5.4%), urinary tract (6.1%), genitourinary (12.2%), head and neck (4.1%), hepatocellular carcinoma (3.0%), breast (2.4%), others (11.1%) |
| **Fanola 2018 [15]** | Prostate (13.7%), breast (6.5%), bladder (7.5%), gastrointestinal (20.5%), lung or pleura (11%), skin (5.9%), pancreatic (3.8%), liver, gallbladder, or bile ducts (3.8%), esophageal (2.5%), oropharyngeal (2.6%), renal (2.5%), uterine (2.1%), brain (2.1%), genital (1.3%), thyroid (1.1%),others (7.8%) |
| **Shah 2018 [18]** | Breast (19.2%), gastrointestinal (12.7%), lung (12.3%), genitourinary (29.2%), gyneco-oncological (2.4%), hematological (9.8%), others (14.4%) |
| **Chen 2019 [14]** | Prostate (28.6%), breast (14.7%), colorectal (16.1%), gastrointestinal (3%), lung (3.1%), melanoma (5.9%), leukemia or lymphoma (5.2%), gynecological (6.6%), genitourinary (12.2%), head and neck (3.9%), thyroid (2.5%), brain (0.3%), others (3%), unspecified cancer type (3.9%) |
| **Sawant 2019 [21]** | N.A. |
| **Yasui 2019 [23]** | Gastrointestinal (44.2%), lung (24.1%), genitourinary (11.2%), head and neck (9.8%), breast (4%), hematological (3.1%), other (3.6%) |
| **Wu 2020 [22]** | N.A. |

**Table S1.** Type and prevalence of cancer in the included studies

| **Author** | **Definition of active cancer** |
| --- | --- |
| **Melloni 2017 [16]** | Recently diagnosed or active cancer or patients who have been treated 6 months prior and were in remission at enrollment |
| **Ording 2017 [20]** | N.A. |
| **Kim 2018 [19]** | Newly diagnosed cancer while taking DOAC for history of non-valvular atrial fibrillation |
| **Fanola 2018 [15]** | New incident cancer or recurrence of remote cancer (> 5 years prior) during study period |
| **Shah 2018 [18]** | Actively treated cancer with chemotherapy, radiation therapy, or cancer surgery within 6 months prior to the start of anticoagulation |
| **Chen 2019 [14]** | Actively treated cancer receiving treatment with hormonal or chemoterapeutic agents |
| **Sawant 2019 [21]** | N.A. |
| **Yasui 2019 [23]** | Evidence of neoplasm on imaging, or ongoing cancer therapy |
| **Wu 2020 [22]** | Cancer receiving treatment or diagnosed within last 6 months |

**Table S2.** Definitions of active cancer

DOACs: direct oral anticoagulant

**Figure S1.** Forest plots showing the comparison between DOACs vs VKAs in patients with cancer and AF. The RRs for efficacy outcomes obtained using a fixed-effects model are shown.

AD: all-cause death; CVD: cardiovascular death; HS: hemorrhagic stroke; IS: ischemic stroke; MI: myocardial infarction; SSE: stroke or systemic embolism.

**Figure S2.** Forest plots showing the comparison between DOACs vs VKAs in patients with cancer and AF. The RRs for safety outcomes obtained using a fixed-effects model are shown.

MB: major bleeding; NMCRB: non-major clinically relevant bleeding; IC: intracerebral; GIB: gastrointestinal bleeding.

**Figure S3.** Forest plots showing the comparison between DOACs vs VKAs in patients with cancer and AF. The RRs for efficacy outcomes obtained pooling data from Retrospective Cohort studies are shown.

AD: all-cause death; CVD: cardiovascular death; HS: hemorrhagic stroke; IS: ischemic stroke; MI: myocardial infarction; SSE: stroke or systemic embolism.

**Figure S4.** Forest plots showing the comparison between DOACs vs VKAs in patients with cancer and AF. The RRs for safety outcomes obtained pooling data from Retrospective Cohort studies are shown.

IC: intracerebral; GIB: gastrointestinal bleeding.

**Figure S5.** Forest plots showing the comparison between DOACs vs VKAs in patients with cancer and AF. The RRs for efficacy outcomes obtained pooling data from Post-hoc Analysis studies are shown.

AD: all-cause death; CVD: cardiovascular death; HS: hemorrhagic stroke; IS: ischemic stroke; MI: myocardial infarction; SSE: stroke or systemic embolism.

**Figure S6.** Forest plots showing the comparison between DOACs vs VKAs in patients with cancer and AF. The RRs for safety outcomes obtained pooling data from Post-hoc Analysis studies are shown.

MB: major bleeding; NMCRB: non-major clinically relevant bleeding; IC: intracerebral; GIB: gastrointestinal bleeding.

**Figure S7.** Forest plots showing the comparison between DOACs vs VKAs in patients with cancer and AF. The RRs for efficacy outcomes obtained pooling data from Propensity-score Matched or Adjusted Analyses are shown.

AD: all-cause death; CVD: cardiovascular death; IS: ischemic stroke; MI: myocardial infarction; SSE: stroke or systemic embolism.

**Figure S8.** Forest plots showing the comparison between DOACs vs VKAs in patients with cancer and AF. The RRs for safety outcomes obtained pooling data from Propensity-score Matched or Adjusted Analyses are shown.

MB: major bleeding; NMCRB: non-major clinically relevant bleeding; IC: intracerebral; GIB: gastrointestinal bleeding.

**Figure S9.** Forest plots showing the comparison between DOACs vs VKAs in patients with cancer and AF. The RRs for efficacy outcomes obtained pooling data about patients with active cancer are shown.

AD: all-cause death; CVD: cardiovascular death; IS: ischemic stroke; MI: myocardial infarction; SSE: stroke or systemic embolism.

**Figure S10.** Forest plots showing the comparison between DOACs vs VKAs in patients with cancer and AF. The RRs for safety outcomes obtained pooling data about patients with active cancer are shown.

MB: major bleeding; NMCRB: non-major clinically relevant bleeding; IC: intracerebral; GIB: gastrointestinal bleeding.
